# Removal of Psoas and Iliac Muscles of the Left Side for Sarcoma

**Published:** 1904-06

**Authors:** Geo. H. Noble

**Affiliations:** Fellow of the American Gynecological Society; Fellow of the American Association of Obstetricians and Gynecologists; Fellow of the Southern Surgical and Gynecological Association; Gynecologist to Grady Hospital, Atlanta, Ga.


### REMOVAL OF PSOAS AND ILIAC MUSCLES OF THE LEFT SIDE FOR SARCOMA.

BY GEO. H. NOBLE, M.D.

Fellow of the American Gynecological Society; Fellow of the American Association of Obstetricians and Gynecologists; Fellow of the Southern Surgical and Gynecological Association; Gynecologist to Grady Hospital, Atlanta, Ga.

On July 5, 1901, I removed the uterus and appendages of Mrs. J. E. F., for adeno-carcinoma of the cervix. The ulceration was confined to the posterior wall of the cervical canal. With this exception the portio-vaginalis looked to be in a fair condition. There were no signs of extension of the disease beyond the limit of the uterus. It, therefore, appeared to be a favorable case for operation.

The progress of the case was very satisfactory for eighteen months after hysterectomy. She then experienced pain in the left inguinal region extending along the anterior crural nerve, and later she discovered three small nodular, inelastic and immovable masses between the anterior superior spinous process of the ilium and the promontory of the sacrum. At this time the left thigh became sharply flexed upon the abdomen, and fixed by contraction of the psoas muscle on that side. The pain was severe, and unless profoundly impressed by morphine she was unable to change her position without suffering, even when assisted by the nurse.

On physical examination the mass above mentioned was easily detected at the bifurcation of the iliac artery on the left side, projecting above the brim of the pelvis, shading off externally into an imperfectly outlined boggy mass, and extending two and a half inches from above downward and outward in the course of the psoas muscle. The femoral pulse, when compared with the other side, was found to be unchanged. The pelvic examination was negative. She complained of nausea, loss of flesh and strength. Temperature 100°; pulse 100.

I advised exploratory operation and removal of the mass to give temporary relief from pain and flexion of thigh.

She returned March 6, 1903 (twenty months after hysterectomy). There was marked loss of flesh, increase of pain and decrease in size of the left thigh and leg. They appeared to be a third smaller than the other side. No measurements, however were made.

The mass had lost its nodular feeling and presented a semi-elastic and boggy sense to the touch. It had increased fully one hundred per cent. in size and extended an inch further externally. The femoral pulse was obliterated, but collateral circulation seemed to be well established. The pelvic examination proved negative. Temperature 100°; pulse 126.

The condition was much worse, and she requested the operation, expressing the wish that she might die under it. So it was undertaken March 11, 1903.

The abdomen was opened for the purpose of exploration, to search for recurrence of malignancy in the deep pelvis, and to ascertain if the intestines were involved in the mass on the pelvic brim. The deep pelvis proved to be absolutely free up to the bifurcation of the iliac vessels, but there the internal iliac glands on the left side seemed to be the center of the growth.

The abdominal and pelvic viscera being free, the incision between the recti muscles was closed and a curved incision, four inches long, was made in the left inguinal region an inch and a half from the ilium. This incision was bisected by a line drawn from the anterior superior spinous process to the umbilicus.

I cut down upon the peritoneum and pushed it off for some distance beyond the extremities of the wound, and inwardly far enough to completely clear the mass and elevate the ureter and hold it out of the way with a long retractor.

Knowing the collateral circulation was established through the internal iliac artery, it was my intention to remove the external iliac with the mass, as there was no pulsation on the distal side, but in my efforts to liberate the internal iliac a softened point in the Y of the bifurcation was lacerated. This required ligation of the internal iliac above, and both its anterior and posterior branches below the point of injury. Very little difficulty was experienced in this step of the operation, even though the work was all extraperitoneal. On account of obstructing the collateral circulation by ligation of the internal iliac artery, it became necessary to preserve the external iliac artery through which some blood must have passed; though it was impossible to detect pulsation below the mass. This vessel was rapidly liberated by a blunt dissection from the bifurcation downward, and as soon as the mass constricting it was removed pulsation began in the femoral artery. The calibre of the external iliac remained diminished about one half.

Dissection of the anterior crural nerve was tedious, the mass having surrounded it completely. However, it was eventually accomplished.

The external iliac vein was entirely obliterated and no collateral circulation could be found above the deep epigastric vein.

After freeing the vessels and nerve and pushing the ureter and colon aside, the bulk of the mass was turned out by blunt dissection; forcing the hand down the iliac fossa behind the psoas and internal muscles, following closely the contour of the bone, separating from below upward, the muscles were severed at Poupart's ligament and the lower end brought out of the wound. Then the fingers easily slipped along the psoas muscle to its origin at the transverse processes of the lumbar spine, from which it was cut by curved scissors.

The wound then appeared like an enormous pocket, and required gauze drainage, as the abdominal viscera was unable to force the peritoneum deep enough to fill it.

The mass proved to be sarcoma*, originating in the iliac glands and extending to the left psoas muscle. The growth of the disease was rapid after involving the muscle, which at the time of the operation began to show signs of breaking down. The muscle was converted into a spindle-shaped cavity, containing about an ounce and a half of peculiar milk-like fluid.

The patient's recovery was slow, but the relief she received by this operation was well worth the undertaking. The pain was relieved and the flexion and fixation of the thigh upon the abdomen was overcome, but she was left with very little power of flexing the limb. This, however, should be expected in such circumstances.

The interesting features are: First, removal of the psoas and iliac muscles; second, occurrence of carcinoma and sarcoma in the same subject; third, extraperitoneal ligation of the anterior and posterior branches of internal iliac artery.

On making inquiry at the Surgeon-General's Library, I was informed that nothing of the kind has ever been done. Therefore, this is, in all probability, the first removal of these muscles.

---

### CANCER RESEARCH ABANDONED.

That the researches in regard to the etiology of cancer have failed miserably is a melancholy fact, but true nevertheless. We are as much in the dark in regard to the cause of this dread disease as we ever were. The New York Senate Committee on Finance has, after spending $100,000, refused to appropriate any more money for the maintenance of the Buffalo Cancer Laboratory, asserting that nothing could be shown for the money expended.

---

**This Journal and Atlanta Semi-weekly Journal one year for $1.25 cash in advance. No checks. Strictly to new subscribers.**

*Though it is difficult to differentiate between carcinomatous metastasis in the lymphal glands and lymphosarcoma, the above specimen was examined by a competent pathologist, who was satisfied with his findings.

